# An ultrasensitive near-infrared ratiometric fluorescent probe for imaging mitochondrial polarity in live cells and *in vivo*†
†Electronic supplementary information (ESI) available: Synthetic procedure for **1–4**; NMR and mass spectra of **1–4**; spectroscopic properties and confocal imaging. See DOI: 10.1039/c5sc04099j


**DOI:** 10.1039/c5sc04099j

**Published:** 2015-11-23

**Authors:** Haibin Xiao, Ping Li, Wei Zhang, Bo Tang

**Affiliations:** a College of Chemistry , Chemical Engineering and Materials Science , Collaborative Innovation Center of Functionalized Probes for Chemical Imaging in Universities of Shandong , Key Laboratory of Molecular and Nano Probes , Ministry of Education , Shandong Provincial Key Laboratory of Clean Production of Fine Chemicals , Shandong Normal University , Jinan 250014 , P. R. China . Email: tangb@sdnu.edu.cn

## Abstract

Mitochondrial polarity is a crucial characteristic of these indispensable organelles, and tremendously impacts cellular events. Herein, we describe a new mitochondria-targeting fluorescent probe **MCY-BF_2_** that is singularly sensitive and specifically responsive to mitochondrial polarity. The pull–push system in the conjugated structure of **MCY-BF_2_** is responsible for the polarity-ultrasensitivity due to the excited state intramolecular charge transfer (ICT). By combining with cardiolipin, **MCY-BF_2_** preferentially accumulates in mitochondria. Because the fluorescence emission wavelengths exhibit an obvious red-shift with increasing media polarity, the fluorescence intensity ratio at two different wavelengths *versus* the solvent dielectric constant can quantify the mitochondrial polarity. Experimental results demonstrate that the fluorescent intensity of **MCY-BF_2_** in a non-polar solvent, dioxane, is 120 times higher than that in a polar solvent, dimethyl sulfoxide. As the first near-infrared (NIR) and most sensitive fluorescent imaging probe for polarity, **MCY-BF_2_** can locate exclusively in mitochondria in various cells and discriminate polarity differences between normal and cancer cells. Also, the intrinsic polarity variance at different developmental stages in *Caenorhabditis elegans* (*C. elegans*) was reported here for the first time. Interestingly, the embryonic development stage has a more non-polar environment with a dielectric constant of 7.20, and in contrast the polarity at the young adult stage changes to 10.07. In addition, *in vivo* imaging results suggest that the tumor tissues of mice have an obviously lower polarity than that in normal tissues. Altogether, the merits of the NIR property, high sensitivity and moderate Stokes shift all greatly promote the accuracy of imaging. This probe will be a promising tool for studying biological processes and the pathological mechanism of polarity-related diseases.

## Abstract 2

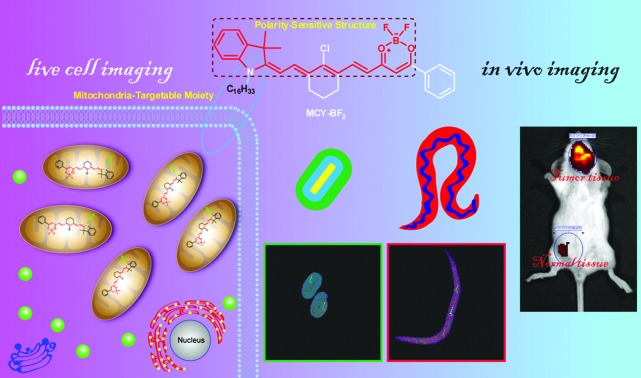
We describe a new mitochondria-targeting fluorescent probe **MCY-BF_2_** that is singularly sensitive and specifically responsive to mitochondrial polarity.

## Introduction

The polarity of a cell is the feedback of a series of complex mechanisms that establish and maintain functionally of particular domains.^1^ Many cellular processes involved in spatial arrangement and protein composition such as differentiation, localized membrane growth, activation of the immune response, directional cell migration, and vectorial transport of molecules across cell layers may lead to changes and development of polarity. Therefore, abnormal changes in polarity are closely linked with disorders and diseases.^2^–^4^ As the powerhouses of cells, mitochondria are vital intracellular organelles and play critical roles in cellular metabolism, including providing metabolic energy by oxidative phosphorylation, a cell signaling platform *via* reactive oxygen species (ROS) production, regulation of Ca^2+^ homeostasis, and triggering of cell apoptosis.^5^–^9^ It is noteworthy that the unique functions of mitochondria are closely related to maintaining homeostasis of their parameters and microenvironments, such as pH,^10^–^12^ viscosity,^13^ polarity,^14^ temperature^15^ and so on. In particular, mitochondrial polarity is a crucial characteristic of these indispensable organelles. Numerous events may be affected by the mitochondrial polarity, including transportation or interaction of proteins, specific activity and stability of proteins or enzymes, and the maintaining of cell function and homeostasis. Thus, to accurately track mitochondrial polarity is of great importance.

Fluorescence imaging is a promising and powerful method for monitoring various bioactive molecules in living systems. Consequently, an increasing number of fluorescent probes suitable for real-time imaging have been developed in recent years, which has facilitated progress in cell biology and therapeutics imaging.^16^–^18^ Nevertheless, ideal fluorescent probes for monitoring mitochondrial polarity should possess the following unique merits: (1) high sensitivity and selectivity to polarity without being affected by other complicated mitochondrial microenvironments; (2) maximum fluorescence excitation and emission wavelengths in the NIR region to reduce interference from background fluorescence; (3) accurate mitochondria-targeting ability, and (4) a ratiometric fluorescence response for a quantitative assay. Although some polarity-sensitive fluorescent probes for solvents, the surface of a protein, and even live cells have been exploited in recent years,^14^,^19^–^27^ a versatile polarity probe satisfying all the aforementioned requirements has not been reported.

**Scheme 1:**
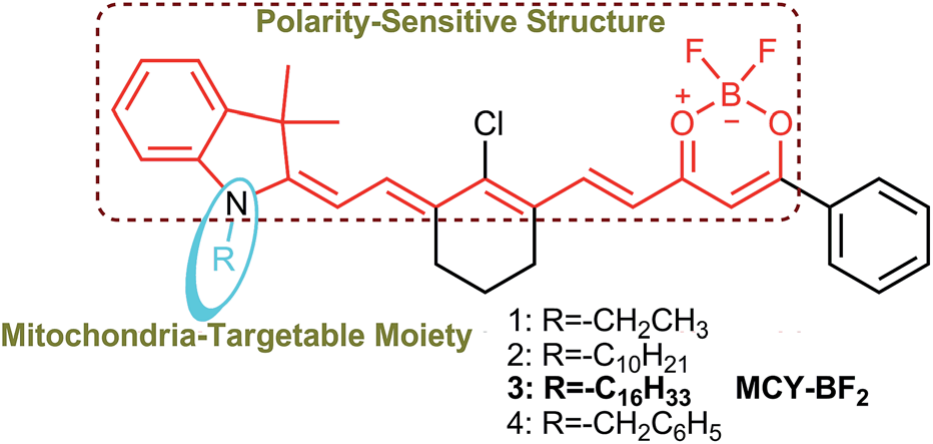
Chemical structures of compounds **1–4** and the corresponding functional units.

To solve these problems mentioned above, we herein fabricated a series of new compounds, **1–4** (Scheme 1), composed of a merocyanine moiety with various lipophilic side chains and a difluoroboronate moiety used to sensitively monitor the polarity. In this framework, the long conjugated system and asymmetric structure account for the NIR excitation/emission spectra and large Stokes shift, respectively. Meanwhile, the tertiary amine and difluoroboronate moieties serve as electron donating and accepting groups, respectively, resulting in the formation of an intense push–pull construction. When the environment polarity increases, the excited state energy can be dissipated due to the dipole–dipole interaction between the probe molecules and solvent due to the excited state ICT,^28^,^29^ which is responsible for the polarity sensitivity. Therefore, the designed compounds should exhibit weak fluorescence and a longer emission wavelength in polar media, and in contrast, a strong fluorescence and shorter emission wavelength in non-polar media.^23^ As a result of red-shifting of the emission wavelength with increasing polarity, a plot of the fluorescence intensity ratios at two different wavelengths *versus* the dielectric constant may be achieved, which can then be utilized to estimate the polarity of certain media and live cells. Considering the characteristics of mitochondria which distinguish them from other subcellular compartments, cardiolipin (CL, a diphosphatidylglycerol lipid) is exclusively found in the inner membrane of mitochondria.^30^ Previous studies revealed that some molecules bearing lipophilic cations can effectively bind mitochondrial CL through electrostatic attraction and lipophilic interactions.^31^ Based on this idea, we introduced various substituents, including ethyl, benzyl, *n*-decane and *n*-hexadecyl groups attached to the nitrogen atom to give four compounds. In one resonance structure of these molecules, there is one positive charge on the nitrogen atom (Scheme S1†). Therefore we speculate that they can grasp CL to target mitochondria specifically.

In this article, we present the synthesis and characterization of four new polarity sensitive compounds. By analyzing their optical properties, we found these compounds could accurately analyse the dielectric constant of the microenvironment. Further comparison with their localization effect shows that a selected compound termed **MCY-BF_2_** bearing an *n*-hexadecyl group displays a better mitochondria-targeting capability than the other three compounds. As the first NIR ratiometric fluorescent probe, it was utilized to discriminate the polarity variance between normal and cancer cells. By means of the fluorescence intensity ratio of 795 and 765 nm, the precise mitochondria polarity in different cells and in *C. elegans* at different developmental stages was also measured. In addition, the polarity difference in normal and tumor s of a mouse was visualized *in vivo*.

## Results and discussion

Compounds **1–4**, as green solids, were synthesized in moderate yield under relatively mild conditions (Scheme S2†). In brief, quaternary ammonium salts containing various lipophilic side chains were reacted with 2-chloro-1-formyl-3-hydroxymethylenecyclohexene to obtain intermediates **5–8**. Subsequently, intermediates **5–8** underwent a condensation reaction with compound **c** to form compounds **1–4**. Full synthetic details and characterization of the new compounds can be found in the ESI.†

**Fig. 1:**
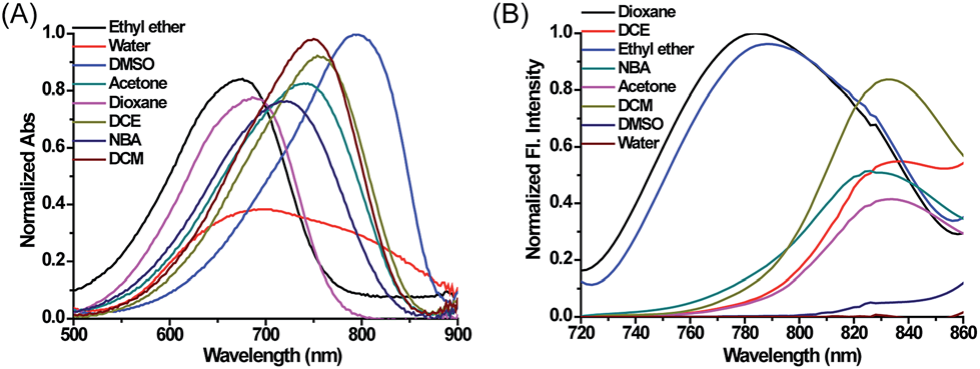
The normalized absorption and fluorescence spectra of **MCY-BF_2_** (10 μM) in eight solvents with different polarity. The excitation wavelength is 700 nm.

With these compounds in hand, we firstly tested their photophysical properties in detail. The absorption and emission profiles of **1–4** in eight common solvents *i.e.* water (dielectric constant *ε* = 80.4), dimethyl sulfoxide (DMSO, *ε* = 48.9), acetone (*ε* = 20.7), *n*-butyl alcohol (NBA, *ε* = 17.8), dichloroethane (DCE, *ε* = 10.4), dichloromethane (DCM, *ε* = 9.14), ethyl ether (*ε* = 4.34) and dioxane (*ε* = 2.21) are displayed in Fig. 1, Fig. S1 and Table S1.† As expected, their maximum emission peaks are all in the NIR range (>750 nm). All the emission spectra of **1–4** show dramatic polarity-dependence and the absorption intensity in all the solvents slightly changes (Fig. 1 and S1†). For example, there is a 120-fold increase in the fluorescence intensity in dioxane (*ε* = 2.21) compared with that in DMSO (*ε* = 48.9) for **MCY-BF_2_** at 800 nm. These phenomena are in accord with the polarity responsive principle.^28^ Accompanied by the increase in solvent polarity, all four compounds exhibit a red-shift in the maximum emission wavelength, from about 780 nm in dioxane to about 830 nm in acetone. Meanwhile, the fluorescence quantum yield decreases noticeably with increasing solvent polarity from about 11% in dioxane to about 1.0% in DMSO (Table S1†).

**Fig. 2:**
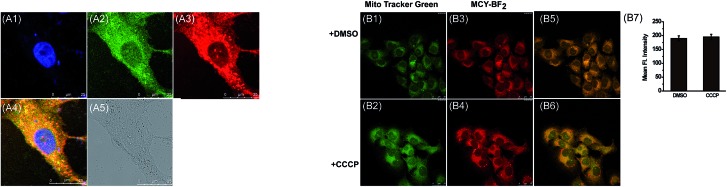
(A1–A5) The co-localization fluorescence images of **MCY-BF_2_** in HepG2 cells. (A1) DAPI (1.0 μg mL^–1^) stain, *λ*_ex_ = 405 nm, collected 410–450 nm. (A2) Mito Tracker Green (0.1 μM) stain, *λ*_ex_ = 488 nm, collected 495–550 nm. (A3) **MCY-BF_2_** (10 μM) stain, *λ*_ex_ = 633 nm, collected 700–800 nm. (A4) Merged image of (A1–A3). (A5) The bright-field image. (B1–B6) Confocal fluorescence images of 4T1 cells stained with Mito-Tracker Green (0.1 μM, *λ*_ex_ = 488 nm, collected 495–550 nm, green channel) and **MCY-BF_2_** (10 μM, *λ*_ex_ = 633 nm, collected 700–800 nm, red channel) in the absence or presence of CCCP (10 μM). (B7) Fluorescence intensity output of **MCY-BF_2_** in images (B3 and B4).

Next we evaluated the mitochondrial localization ability of the four compounds. Firstly, co-localization imaging experiments were performed in living mouse mammary carcinoma 4T1 cells simultaneously loaded with these compounds and Mito Tracker Green, a commercial mitochondrial dye. As shown in Fig. S2,† all four compounds can readily penetrate into 4T1 cells. By comparing the co-localization effects, **MCY-BF_2_** showed apparently superior mitochondria-targeting ability to compounds **1**, **2** and **4**. In addition, a similar targeting ability of **MCY-BF_2_** was testified in human hepatoma cells (HepG2) by means of co-localization experiments using Mito Green Tracker (Fig. 2A) and 5,5′,6,6′-tetrachloro-1,1′,3,3′-tetraethylbenzimidazolcarbocyanine iodide (JC-1, Fig. S3†), with a co-localization coefficient of 0.91 and 0.92, respectively. Other subcellular compartment stain experiments using **MCY-BF_2_** and the corresponding commercial organelle-specific dye were then carried out (Fig. S4†). Inside lysosomes (Lyso), endoplasmic reticulum (ER), and Golgi apparatus (Golgi), significantly smaller co-localization coefficient values were found. These results demonstrate that **MCY-BF_2_** can preferentially accumulate in mitochondria. To further verify the mitochondria-targeting mechanism, 4T1 cells treated with the membrane-potential uncoupler 3-chlorophenylhydrazone (CCCP) that can collapse the mitochondrial membrane potential were simultaneously incubated with **MCY-BF_2_** and Mito-Tracker Green. In this experiment, we found that the fluorescence brightness of **MCY-BF_2_** was consistent in the absence or presence of CCCP (Fig. 2B3 and B4). Moreover, the mitochondria-targeting effect of **MCY-BF_2_** is almost unchanged upon treatment with CCCP (Fig. 2B5 and B6). The above results implied that **MCY-BF_2_** might locate in mitochondria in live cells *via* anchoring CL due to the lipophilic cation group bearing long aliphatic chains, and is independent of the mitochondrial membrane potential. Also, Fig. S5† suggests that **MCY-BF_2_** has low cytotoxicity. On the basis of the above selectivity results, we draw the conclusion that the selected compound **MCY-BF_2_** is a new fascinating probe endowed with polarity sensitivity and excellent mitochondria-targeting ability.

**Fig. 3:**
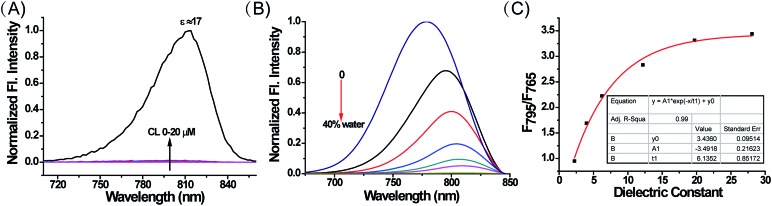
(A) The fluorescence spectra of **MCY-BF_2_** (10 μM, *ε*; = 80.4) upon addition of CL (0–20 μM) and its fluorescence spectra in a weaker polarity environment (*ε* = 17) with 20 μM CL. (B) The fluorescence spectra of **MCY-BF_2_** (10 μM) in dioxane–water mixtures (water from 0 to 40%). (C) The fluorescence intensity ratio of 795 and 765 nm with the dielectric constant. The excitation wavelength is 650 nm.

Subsequently, we investigated whether CL could influence the fluorescence of **MCY-BF_2_**, since it accumulates in mitochondria in combination with CL. As illustrated in Fig. 3A, upon addition of CL from 0 to 20 μM *i.e.*, the physiological range of CL in mitochondrial membranes,^32^,^33^ the fluorescence intensity of **MCY-BF_2_** (10 μM, *ε* = 80.4) increased slightly. In contrast, **MCY-BF_2_** showed intense fluorescence in a low polarity environment (10 μM, *ε* = 17) in the presence of 20 μM CL. These results validate that **MCY-BF_2_** was not affected by CL. In addition, interference experiments showed that the fluorescence signal of **MCY-BF_2_** changed very little in the presence of various ROS, nucleophilic thiols, amino acids, different pH buffers and viscosity (Fig. S6–S8†). Further experiments showed that **MCY-BF_2_** had high photostability (Fig. S9†).

To accurately analyze the mitochondrial polarity in living cells and *in vivo* using **MCY-BF_2_**, the curve of the quantitative relationship between the fluorescence intensity ratio and polarity should be examined. For this purpose, we investigated the emission of **MCY-BF_2_** in mixtures of dioxane and water with different proportions of water from 0 to 40% to represent the rise in polarity.^34^ Fig. S10A† indicates that the absorbance of **MCY-BF_2_** in mixtures of water from 0 to 40% is almost unchanged, whereas, as illustrated in Fig. 3B, the emission maximum wavelength is red-shifted from about 780 nm in dioxane to about 820 nm in 40% water. Notably, there is an approximately 45-fold enhancement in the fluorescence intensity at 780 nm when decreasing the polarity of the solvent from a dielectric constant of 28.1 (40% water) to 2.21 (0% water). The relative fluorescence quantum yield was determined to be 11% in 0% water and 1.1% in 40% water (Fig. S10B†). All the results suggest that **MCY-BF_2_** is extremely sensitive to polarity changes. More importantly, to the best of our knowledge, this is the most sensitive near-infrared fluorescent probe for polarity up to now. By plotting the fluorescence intensity ratio *F*_795_/*F*_765_ against the dielectric constant (Fig. 3C), a calibration curve was obtained with a relationship coefficient of *R*^2^ = 0.99. Thus, it can be used to measure the polarity of certain media or live cells using confocal fluorescence imaging in view of the collection window up to a maximum wavelength of 800 nm. Moreover, Fig. 3C reveals that the *F*_795_/*F*_765_ ratio increases dramatically from 0.8 to 3.0 with increasing solvent polarity when the dielectric constant is below 15, then the change tends to be gradual when the solvent dielectric constant is above 20, indicating that **MCY-BF_2_** is extraordinarily sensitive to weak polarity media. Taken together, **MCY-BF_2_** is an excellent near-infrared fluorescent probe that can be used to exclusively quantify polarity fluctuations based on changes in its fluorescence intensity ratio *F*_795_/*F*_765_.

**Fig. 4:**
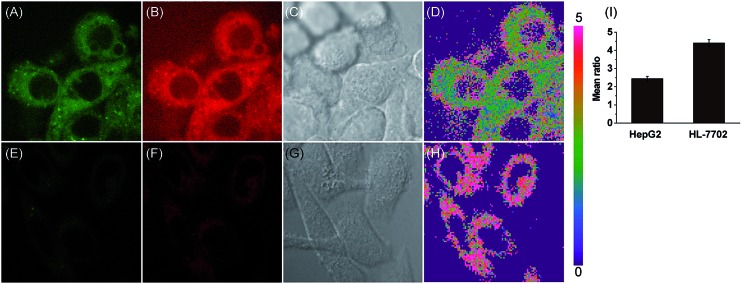
Ratiometric fluorescence imaging of the mitochondrial polarity in HepG2 (A–D) and HL-7702 (E–H) cells stained with **MCY-BF_2_** (10 μM). (A) and (E) were green channels collected at 760–770 nm. (B) and (F) were red channels collected at 790–800 nm. (C) and (G) are bright-field images. (D) is the ratiometric image between images (B) and (A), and (H) is ratiometric image between images (F) and (E). (I) is the output of the mean ratio in images (D) and (H).

Accumulating evidence suggests that normal and cancer cells display microenvironment differences,^35^–^37^ prompting us to exploit the biological feasibility of **MCY-BF_2_** as a ratiometric fluorescence imaging probe. We intend to quantitatively detect mitochondrial polarity in cells using confocal fluorescence imaging in different cells. HepG2 cells and normal human liver cells (HL-7702) were incubated with **MCY-BF_2_** (10 μM), and two fluorescence channels, green for 760–770 nm and red for 790–800 nm, were collected. As illustrated in Fig. 4, the red fluorescence intensity (Fig. 4B and F) was obviously higher. Fig. 4D and H show the fluorescence intensity ratio between the red and green channels, in which a mean ratio was 2.44 ± 0.13 (Fig. 4I) in HepG2 cell mitochondria. The corresponding dielectric constant was 7.72 ± 0.32 according to the fluorescence intensity ratio curve in Fig. 3C. The mean ratio in HL-7702 cell mitochondria is 4.40 ± 0.19, which indicates that the corresponding dielectric constant should be more than 30. Similar results are obtained in 4T1 cells and normal human mammary epithelial cells MCF-10A (Fig. S11†). This demonstrates that polarity in cancer cells is lower than that in normal cells.^38^–^40^ All these results substantiated that **MCY-BF_2_** can serve as a highly sensitive ratiometric fluorescence imaging probe to quantitatively indicate the mitochondrial polarity within live cells.

**Fig. 5:**
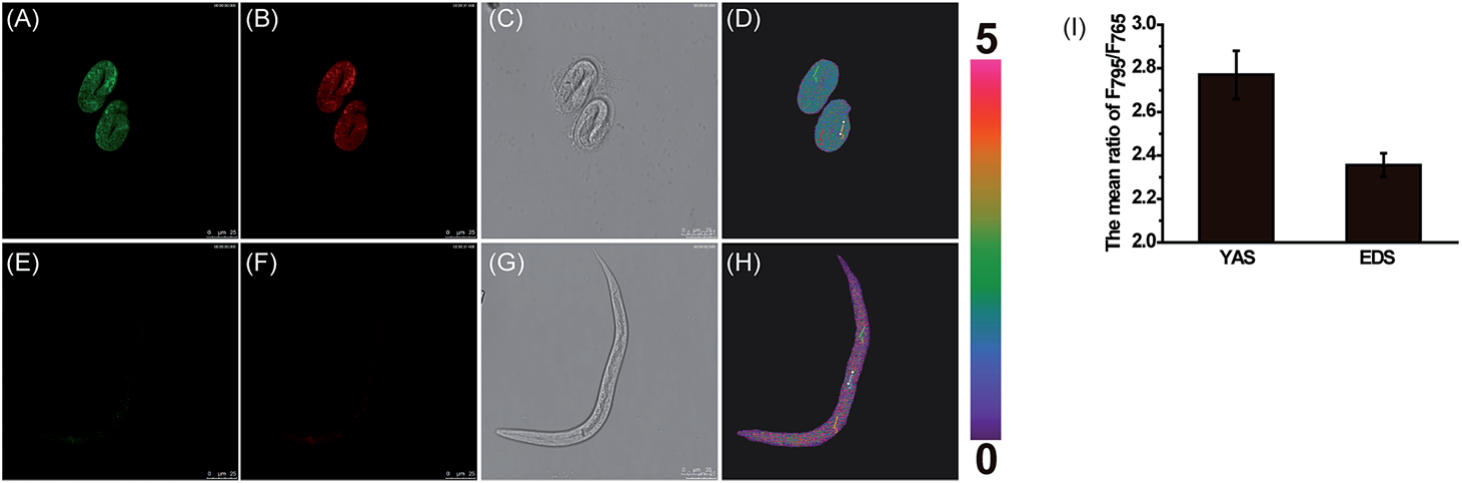
Confocal ratiometric fluorescence imaging of the mitochondrial polarity at EDS (A–D) and YAS (E–H) of *C. elegans* stained with **MCY-BF_2_** (20 μM). *λ*_ex_ = 633 nm, collected at 760–770 nm for (A) and (E), and collected at 790–800 nm for (B) and (F). (D) is the ratiometric fluorescence imaging between (B) and (A), and (H) is that between (F) and (E). (C) and (G) are bright-field images, and (I) is the mean ratio from images (D) and (H) (*n* = 3).

Previous studies speculate that the polarity may change continuously in the *C. elegans* development process.^41^,^42^ To explore whether these inherent polarity differences exist, we used **MCY-BF_2_** to measure the polarity by *in vivo* visualization of *C. elegans* at various development stages. *C. elegans* at embryonic development stage (EDS) and young adult stage (YAS) were incubated with **MCY-BF_2_** (20 μM) for 30 min, after which confocal fluorescence imaging was performed immediately without washing the incubation solution. As shown in Fig. 5, **MCY-BF_2_** can fluoresce well in *C. elegans* both at EDS and YAS. Interestingly, its fluorescence at EDS (Fig. 5A and B) was remarkably brighter than that at YAS (Fig. 5E and F). To further quantify the precise polarity of *C. elegans* at EDS and YAS, we investigated the fluorescence ratio based on two groups of confocal fluorescence images captured with *λ*_em_ = 760–770 nm and *λ*_em_ = 790–800 nm. As shown in Fig. 5, the ratio of the two channels at EDS (Fig. 5D) is obviously smaller than that at YAS (Fig. 5H). The mean ratio value of *C. elegans* at EDS is 2.36 ± 0.05, which corresponds to a dielectric constant of 7.20 ± 0.19. Meanwhile, the mean ratio at YAS is 2.76 ± 0.11, indicating a dielectric constant of 10.07 ± 0.29 (Fig. 5I). This is the first time it has been confirmed that the polarity of *C. elegans* at EDS is smaller than that at YAS.

**Fig. 6:**
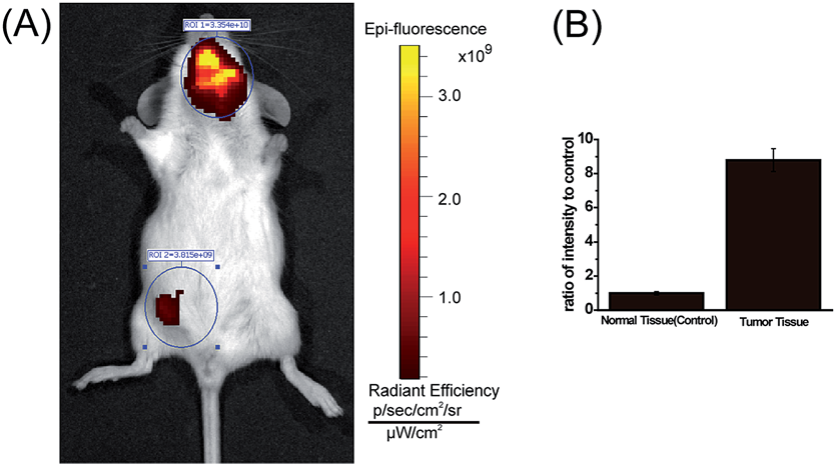
(A) Representative fluorescent images (pseudocolor) of *in vivo* normal and tumor tissues with **MCY-BF_2_** (*n* = 3). (B) Fluorescence emission intensity ratios to the control groups. The excitation filter was 740 nm, and the emission filter was 780 nm.

One of major advantages of the new **MCY-BF_2_** probe over existing polarity-sensitive probes is that it has both absorption and emission in the NIR region. We consider **MCY-BF_2_** is well suited for biological imaging in live animals because NIR light leads to minimum photodamage to biological samples, deep tissue penetration, and reduced interference from background autofluorescence by biomolecules in living animals.^43^–^45^ Thus, a further effort was made to distinguish the polarity variance of normal and tumor tissues in live small animals. We constructed a mouse model of mammary carcinoma for *in vivo* imaging. Specifically, mammary carcinoma 4T1 cells were inoculated into the neck of mice, and after 10 days a tumor mass was obtained. Subsequently, **MCY-BF_2_** (2 × 10^–5^ M in PBS containing 1% DMSO, 100 μL) was injected into the tumor-bearing and normal abdomen tissue of the mice, followed by *in situ* imaging using IVIS Lumina III without shaving the mouses’ skin. The neck tumor mass exhibited a significantly stronger fluorescence (pseudocolor) than that of the normal tissue in the abdomen. The neck tumor tissue displayed an approximately 9-fold higher fluorescence intensity than that of normal tissue (Fig. 6B). We investigated that hypoxia had no effect on the fluorescence of **MCY-BF_2_** (Fig. S12†), further confirming that lower polarity in tumor tissue indeed causes intense fluorescence. These results demonstrate that **MCY-BF_2_** is a prominent fluorescent probe for imaging polarity differences *in vivo*.

## Conclusion

In conclusion, we have developed a new near-infrared ratiometric fluorescent probe, **MCY-BF_2_**, for ultrasensitive sensing of mitochondrial polarity. It has both absorption and emission in the NIR region as well as a modest Stokes shift and is insensitive to various ROS, thiols, amino acids, oxygen, pH values and viscosity. Also, it exhibits good photostability and low cytotoxicity. **MCY-BF_2_** can effectively stain mitochondria by anchoring CL in the inner membrane of mitochondria independent of the mitochondrial membrane potential. It was successfully utilized to distinguish cancer cell and normal cell polarity differences of mitochondria. Moreover, the fluorescence intensity ratio *F*_795_/*F*_765_ at different developmental stages of *C. elegans* showed that the embryonic development stage has a less polar microenvironment with a dielectric constant of 7.20 ± 0.19, while the young adult stage has a more polar microenvironment with a dielectric constant of 10.07 ± 0.29. Furthermore, **MCY-BF_2_** was successfully applied to monitor the polarity distinction in normal and tumor tissues in live animals. To the best of our knowledge, this is the first near-infrared and most sensitive ratiometric fluorescent probe for imaging polarity up to now. Our present work may provide a new method to quantitatively study the polarity both in normal or cancer cells and *in vivo*. Furthermore, it paves the way to elucidating biological processes and pathological mechanisms of mitochondrial polarity-related diseases.

## Supplementary Material

Supplementary information
