# Supplementary material for: An ultrasensitive near-infrared ratiometric fluorescent probe for imaging mitochondrial polarity in live cells and in vivo
Source: Chem Sci. 2015 Nov 23;7(2):1588–93. doi: 10.1039/c5sc04099j (PMC5964966; doi:10.1039/c5sc04099j)
Supplement: Supplementary file 1 [file SC-007-C5SC04099J-s001.pdf]

Electronic Supplementary Material (ESI) for Chemical Science.
This journal is © The Royal Society of Chemistry 2015

Supporting Information

An ultrasensitive near-infrared ratiometric fluorescent probe for imaging mitochondrial polarity in live cells and in vivo

Haibin Xiao,‡ Ping Li,‡ Wei Zhang and Bo Tang*

College of Chemistry, Chemical Engineering and Materials Science, Collaborative Innovation Center of Functional-ized Probes for Chemical Imaging in Universities of Shandong, Key Laboratory of Molecular and Nano Probes, Ministry of Education, Shandong Provincial Key Laboratory of Clean Production of Fine Chemicals, Shandong Normal University, Jinan 250014, P. R. China

*E -mail: tangb@sdnu.edu.cn

Table of Contents

Materials and instruments

Determination of the relative fluorescence quantum yield

Cells and *C. elegans* culture

Instructions for imaging experiments

Scheme S1 The resonance structures of compounds **1-4**

Scheme S2 The synthesis of compounds **1-4**

Table S1 The photophysical properties of **1-4** in eight solvents

Fig. S1 The normalized absorption and fluorescence spectra of compounds **1, 2, 4** in eight solvents

Fig. S2 Confocal fluorescence images of 4T1 cells stained with **1-4** and **MitoTracker Green**

Fig. S3 Confocal fluorescence images of HepG2 cells stained with **MCY-BF$_2$** and **JC-1**

Fig. S4 Confocal fluorescence images of HepG2 cells stained with **MCY-BF$_2$** and commercial organelle-specific dyes

Fig. S5 Cytotoxicity assay

Fig. S6 The fluorescence intensity of **1-4** in the presence of various ROS, thiol and amino acids

Fig. S7 The absorption and fluorescence spectra of **MCY-BF$_2$** under different pH values

Fig. S8 The fluorescence intensity of **MCY-BF$_2$** in methanol- glycerol system under different viscosity

Fig. S9 The photostability experiments of **MCY-BF$_2$**

Fig. S10 The absorption spectra and relative fluorescence quantum yield of **MCY-BF$_2$** in dioxane-water mixtures

Fig. S11 The mitochondrial polarity determination in 4T1 and MCF-10A cells

Fig. S12 The fluorescence response of **MCY-BF$_2$** to oxygen

Materials and instruments

Unless otherwise stated, all reagents were purchased from commercial suppliers and used without further purification. The solvents were purified by conventional methods before use. JC-1, ER-Tracker Red, Lyso-Tracker Green and Mito-Tracker Green were purchased from Invitrogen (USA). Golgi-Tracker Red was purchased from Beyotime Biotechnology. Silica gel (200-300 mesh) used for flash column chromatography was purchased from Qingdao Haiyang Chemical Co., Ltd. **MCY-BF$_2$** was dissolved in dimethyl sulfoxide (DMSO) to produce 1 mM stock solutions. ^{1}HNMR and ^{13}CNMR spectra were determined by 400 MHz (or 300 MHz) and 100 MHz using Bruker NMR spectrometers. The mass spectra were obtained by Bruker maxis ultra-high resolution-TOF MS system. The fluorescence spectra measurements were performed using FLS-920 Edinburgh fluorescence spectrometer. Fluorescence imaging in cells and *C. elegans* were performed with Leica TCS SP5 Confocal Laser Scanning Microscope. All the cells were purchased from Cell Bank of the Chinese Academy of Sciences (Shanghai, China). The KM mice were purchased from Shandong University Laboratory Animal Center. All the animal experiments were carried out in accordance with the relevant laws and guidelines issued by the Ethical Committee of Shandong University.

Determination of the relative fluorescence quantum yield [1,2]

Fluorescence quantum yield of compouds **1-4** was determined by using ICG (Φ_f = 0.13 in DMSO) as a fluorescence standard.

Cells and *C. elegans* culture

HepG2 and MCF-10A cells were cultured in high glucose DMEM (4.5 g of glucose/L) supplemented with 10% fetal bovine serum, 1% penicillin, and 1% streptomycin at 37 °C in a 5% CO_2 /95% air incubator MCO-15AC (SANYO, Tokyo, Japan). One day before imaging, the cells were detached and were replanted

on glass-bottomed dishes. 4T1 and HL-7702 cells were cultured in RPMI 1640. *C. elegans* were cultured at 20 °C on solid nematode growth media (NGM) with Escherichia coli strain OP50. Eggs were placed on the plates (100–200 eggs per plate), and left to hatch and grow to the beginning of the adult stage. All compounds were added into the NGM media every two days and worms were moved to new assay plates every 2 days.

Instructions for imaging experiments

The confocal fluorescence imaging experiments were performed on Leica TCS SP5 Confocal Laser Scanning Microscope. The laser power of confocal imaging is 15 mW (488 nm laser), 5 mW (543 nm laser) and 5 mW (633 nm laser), respectively. The cells and *C. elegans* were incubated with probes and corresponding organelle-specific dyes for 30 min. After the incubation solution was removed, the cells were washed three times with PBS and then imaged. **MCY-BF$_2$** in live cells and *C. elegans* was excited with a 633 nm laser, and two groups of channels were collected (760-770 nm for green channel, 790-800 for red channel). The ratiometric imaging was obtained between red channel and green channel. In vivo imaging was performed on IVIS Lumina III system (Xenogen, USA) with a metal halide lamp (150 W). The mice with tumor mass were anesthetized by an i.p. injection of 4% chloral hydrate (0.25 ml). Then, the mice were injected **MCY-BF$_2$** (20 μM, 100 μL) to normal and tumor tissues and then imaged without shaving the mouse skin. The excitation filter was 740±20 nm, and the emission filter was 790±20 nm.

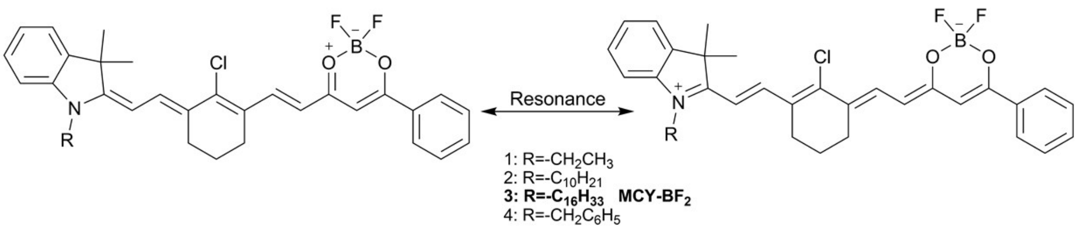

Scheme S1 The resonance structures of compounds **1-4**

Synthesis of compounds 1-4

Compounds **a**, **b** and **c** were synthesized as previous report.[3,4]

Compounds **5-8** were synthesized according to our previous work with minor revision.[5]

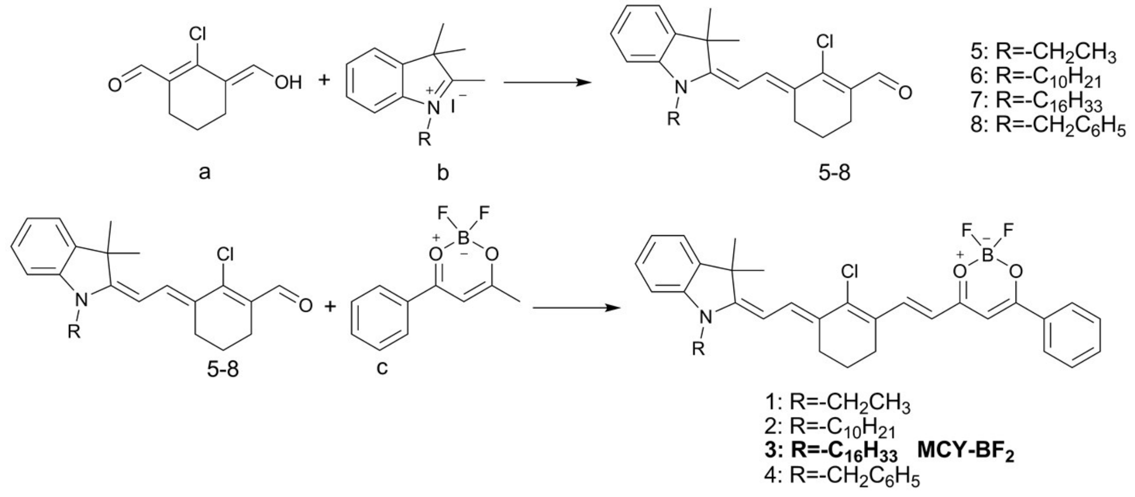

Scheme S2 The synthesis of compounds **1-4**

Synthesis of 5-8

A mixture of 2-chloro-1-formyl-3-hydroxymethylenecyclohexene **a** and corresponding quaternary ammonium salts **b** in toluene were placed in a flask containing acetic acid, and the mixture was stirred at 80 °C under nitrogen atmosphere for 5 h. The crude product was purified by silica gel flash chromatography using CH_2Cl_2:CH_3OH (50:1) as eluent to give compounds **5-8** as fuchsia solid.

Compound **5**: Yield 63%. ^{1}H NMR (300 MHz, $CDCl_3$) 1.29 (t, J=6.9 Hz, 3H), 1.65 (s, 6H), 1.76-1.80 (m, 2H), 2.49 (t, J=6.0 Hz, 2H), 2.59 (t, J=6.0 Hz, 2H), 3.770 (q, J=6.9 Hz, 2H), 5.53 (d, J=12.9 Hz, 1H), 6.74 (d, J=7.8 Hz, 1H), 6.95 (t, J=7.8 Hz, 1H), 7.22 (t, J=7.8 Hz, 2H), 7.86 (d, J=12.9 Hz, 1H), 10.25 (s, 1H). ^{13}CNMR (100 MHz, $CDCl_3$) 11.23, 18.19, 20.95, 24.58, 26.69, 28.35, 29.70, 46.62, 92.44, 106.80, 120.91, 121.87, 123.00, 127.91, 128.47, 131.45, 139.36, 148.82, 161.75, 190.81, 194.83. HRMS (ESI) m/z calcd. for $C_{21}H_{24}ClNO$ [M+H$^+$]: 342.1546, found 342.1561.

Compound **6**: Yield 59%. ^{1}HNMR (400 MHz, $CDCl_3$) 0.88 (s, 3H), 1.27-1.42 (m, 16H), 1.65 (s, 6H), 1.71-1.77 (m, 2H), 2.48-2.57 (m, 4H), 3.67 (s, 2H), 5.54 (d, J=12 Hz, 1H), 6.72 (d, J=6.8 Hz, 1H), 6.95 (d, J=6.8 Hz, 1H), 7.20 (d, J=6.8 Hz, 2H), 7.86 (d, J=12 Hz, 1H), 10.23 (s, 1H) ^{13}CNMR (100 MHz, $CDCl_3$) 14.11,

17.03, 20.97, 22.67, 24.30, 24.60, 26.23, 26.68, 27.11, 28.36, 29.28, 29.35, 29.53, 31.88, 42.65, 46.57, 92.86, 107.09, 120.89, 121.77, 122.80, 127.88, 128.35, 131.56, 139.30, 144.06, 148.82, 162.30, 190.70. HRMS (ESI) m/z calcd. for $C_{29}H_{40}ClNO$ [M+H$^+$]: 454.2871, found 454.2881.

Compound **7**: Yield 55%. ^{1}HNMR (400 MHz, $CDCl_3$) 0.79 (s, 3H), 1.16 (s, 28H), 1.55 (s, 6H), 2.39 (d, J=24 Hz, 6H), 3.56 (s, 2H), 5.41 (d, J=6.0 Hz, 1H), 6.60 (s, 1H), 6.83 (s, 1H), 7.08 (s, 2H), 7.73 (d, J=6.0 Hz, 1H), 10.16 (s, 1H); ^{13}CNMR (100 MHz, $CDCl_3$) 14.16, 21.06, 22.73, 24.64, 25.25, 25.72, 27.14, 28.38, 29.40, 29.51, 29.62, 29.67, 29.73, 31.96, 42.66, 46.57, 92.87, 107.07, 120.89, 121.77, 122.84, 125.56, 127.90, 128.43, 131.43, 139.30, 144.09, 148.68, 162.20, 190.61. HRMS (ESI) m/z calcd. for $C_{35}H_{52}ClNO$ [M+H$^+$]: 538.3810, found 538.3804.

Compound **8**: Yield 56%. ^{1}HNMR (400 MHz, $CDCl_3$) 1.71 (s, 6H), 1.87-1.99 (m, 2H), 2.42-2.60 (m, 4H), 4.91 (s, 2H), 5.52 (d, J=7.2 Hz 1H), 6.71 (d, J=7.2 Hz 1H), 6.97 (s, 1H), 7.18-7.44 (m, 7H), 7,81 (s, 1H), 10.23 (s, 1H) ^{13}CNMR (100 MHz, $CDCl_3$) 20.84, 24.52, 26.55, 28.48, 29.70, 32.33, 46.43, 93.85, 107.15, 121.15, 121.91, 123.82, 126.36, 127.61, 128.02, 128.98, 130.85, 134.11, 135.68, 139.09, 144.35, 148.59, 162.03, 190.36, 190.89, 197.25. HRMS (ESI) m/z calcd. for $C_{26}H_{26}ClNO$ [M+H$^+$]: 404.1776, found 404.1786.

Synthesis of compounds 1-4

Compound **5-8** and **c** were added carefully to acetic anhydride. Sodium acetate was added to the mixture, and the reaction was stirred at 60 °C under nitrogen atmosphere for 3 h. After that, it was poured into 100 mL of saturated $NaHCO_3$ solution and mixed carefully, and then the oil solid was collected with dichloromethane after the aqueous solution was poured off. The organic layers were dried over Na_2SO_4, and evaporated under reduced pressure. Compounds **1-4** were obtained as green solid by column chromatography on silica gel flash chromatography using CH_2Cl_2.

Compound **1**: Yield 47%. ^{1}H NMR (400 MHz, $CDCl_3$): 1.289 (t, J=4.4 Hz, 3H), 1.654 (s, 6H), 1.860 (m,

2H), 2.509-2.570 (m, 4H), 3.770 (q, J=4.4 Hz, 2H), 5.582 (d, J=8.8 Hz, 1H), 6.122 (d, J=9.6 Hz, 1H), 6.489 (s, 1H), 6.580 (d, J=11.2 Hz, 1H), 6.740 (d, J=5.6 Hz, 1H), 6.983 (t, J=4.8 Hz, 1H), 7.517 (t, J=5.2 Hz, 1H), 7.556 (t, J=4.8 Hz, 1H), 7.833 (d, J=8.8 Hz, 1H), 7.994 (d, J=5.2 Hz, 2H), 8.041 (t, J=5.2 Hz, 2H), 8.542 (d, J=9.6 Hz, 1H) ^{13}C NMR (100 MHz, $CDCl_3$): 11.34, 21.13, 26.19, 27.00, 28.29, 37.42, 47.05, 94.14, 97.51, 97.55, 105.10, 107.24, 117.10, 121.58, 121.99, 124.82, 127.08, 127.10, 128.22, 129.50, 132.89, 133.67, 133.70, 134.92, 138.64, 139.80, 143.20, 145.83, 162.96, 177.82, 180.15. HRMS (ESI) m/z calcd. for $C_{31}H_{31}BF_2ClNO_2$ [M+H$^+$]: 534.2183, found 534.2172

Compound **2**: Yield 44%. ^{1}HNMR (400 MHz, $CDCl_3$) 0.89 (s, 3H), 1.27-1.44 (m, 16H), 1.68 (s, 6H), 1.89 (s, 2H), 2.57 (s, 4H), 3.72 (s, 2H), 5.63 (d, J=17.2 Hz, 1H), 6.19 (d, J=20 Hz, 1H),6.52 (s, 1H), 6.77 (d, J=10.4 Hz, 1H), 6.99 (t, J=9.6 Hz, 1H), 7.21-7.24 (m, 2H), 7.49-7.52 (m, 2H), 7.89 (d, J=18.4 Hz, 2H), 8.03-8.06 (m, 2H), 8.63 (d, J=20 Hz 1H) ^{13}CNMR (100 MHz, $CDCl_3$). 13.08, 20.12, 21.64, 23.76, 25.14, 25.38, 26.10, 27.32, 28.25, 28.30, 28.46, 28.50, 28.68, 30.86, 41.83, 46.02, 93.48, 96.39, 96.44, 105.27, 106.50, 109.53, 115.99, 120.54, 120.91, 123.70, 126.04, 126.90, 127.13, 127.82, 127.99, 128.18, 132.64, 133.13, 134.42, 138.73, 142.70, 144.84, 145.02. HRMS (ESI) m/z calcd. for $C_{39}H_{47}ClBF_2NO_2$ [M+H$^+$]: 646.3436, found 646.3446.

Compound **3**: Yield 41%. ^{1}HNMR (400 MHz, $CDCl_3$) 0.88 (s, 3H), 1.25 (s, 28H), 1.66 (s, 6H), 2.41-2.56 (m, 6H), 3.70 (s, 2H), 5.58 (d, J=12 Hz, 1H), 6.12 (d, J=12 Hz, 1H), 6.50 (s, 1H), 6.76 (s, 1H), 6.99 (s, 1H), 7.46-7.55 (m, 4H), 7.84 (d, J=12 Hz, 1H), 8.01-8.05 (m, 3H), 8.54 (d, J=12 Hz, 1H). ^{13}CNMR (100 MHz, $CDCl_3$) 14.13, 21.13, 22.70, 26.17, 26.41, 26.98, 27.12, 28.35, 29.37, 29.50, 29.59, 29.64, 29.70, 31.94, 42.87, 47.05, 94.58, 97.55, 107.56, 116.98, 121.58, 121.92, 124.79, 127.06, 127.98, 128.13, 128.83, 129.03, 129.19, 132.89, 133.63, 134.20, 135.43, 139.71, 143.71, 145.78, 146.04, 163.53, 177.65, 180.05. HRMS (ESI) m/z calcd. for $C_{45}H_{59}ClBF_2NO_2$ [M+H$^+$]: 730.4376, found 730.4341.

Compound **4**: Yield 45%. ^{1}HNMR (400 MHz, $CDCl_3$) 1.74 (s, 6H), 1.78-1.83 (m, 2H), 2.43-2.49 (m, 4H),

4.94 (s, 2H), 5.61 (d, J=17.2 Hz, 1H), 6.19 (d, J=20 Hz, 1H), 6.52 (s, 1H), 6.75 (d, J=10.8 Hz, 1H), 7.01 (t, J=10.0 Hz, 1H), 7.17-7.24 (m, 4H), 7.35-7.41 (m, 2H), 7.49 (t, J=10.4 Hz, 2H), 7.60 (t, J=10.0 Hz, 2H), 7.84 (d, J=17.2 Hz, 1H), 8.05 (d, J=10.0 Hz, 2H), 8.61 (d, J=20 Hz, 1H) ^{13}CNMR (100 MHz, $CDCl_3$) 20.00, 25.01, 25.77, 27.46, 28.49, 45.52, 45.96, 94.25, 96.51, 106.50, 116.63, 120.68, 121.00, 124.48, 125.32, 126.50, 126.70, 127.06, 127.20, 127.84, 128.02, 131.77, 132.20, 132.79, 134.48, 138.39, 143.02, 144.59, 144.76, 161.93, 177.31, 179.30. HRMS (ESI) m/z calcd. for $C_{36}H_{33}ClBF_2NO_2$ [M+H$^+$]: 596.2261, found 596.2271

Table S1 The photophysical properties of **1-4** (10 μM) in eight solvents. Superscript a, b and c represent the dielectric constant of solvents, relative fluorescence quantum yield and molar extinction coefficient, respectively.

(1)

Solvent	ε^a	λ_{abs}/nm	λ_{em}/nm	Stokes shift/nm	ε^c/M^{-1} cm^{-1}	Φ_f^b
H_2O	80.4	664	--	--	22000	<0.001
DMSO	48.9	792	850	58	62000	0.7%
Acetone	20.7	736	830	94	59400	2.6%
NBA	17.8	714	832	118	52400	4.1%
DCE	10.4	754	834	80	66200	4.6%
DCM	9.14	744	834	90	64600	6.0%
Ethyl ether	4.34	668	786	118	58200	10.3%
Dioxane	2.21	688	788	100	54800	11.0%

(2)

Solvent	ε^a	λ_{abs}/nm	λ_{em}/nm	Stokes shift/nm	ε^c/M^{-1} cm^{-1}	Φ_f^b
H_2O	80.4	702	--	--	12400	<0.001
DMSO	48.9	802	850	48	31200	1.0%
Acetone	20.7	744	834	90	27000	3.3%
NBA	17.8	714	830	116	24000	4.7%
DCE	10.4	754	836	82	29200	6.0%
DCM	9.14	744	832	88	29800	7.0%
Ethyl ether	4.34	676	784	108	34000	10.0%
Dioxane	2.21	686	788	102	25000	10.9%

(3, MCY-BF_2)

Solvent	ε^a	λ_{abs}/nm	λ_{em}/nm	Stokes shift/nm	ε^c/M^{-1} cm^{-1}	Φ_f^b
H_2O	80.4	700	--	--	33800	<0.001
DMSO	48.9	792	852	60	88200	0.8%
Acetone	20.7	740	834	94	72800	2.6%
NBA	17.8	716	836	120	67400	3.9%
DCE	10.4	754	840	86	81400	4.3%
DCM	9.14	750	832	82	86600	5.6%
Ethyl ether	4.34	676	780	104	74200	10.5%
Dioxane	2.21	686	788	102	68600	11.0%

(4)

Solvent	ε^a	λ_{abs}/nm	λ_{em}/nm	Stokes shift/nm	ε^c/M^{-1} cm^{-1}	Φ_f^b
H_2O	80.4	674	--	--	19200	<0.001
DMSO	48.9	774	844	70	47400	1.1%
Acetone	20.7	714	834	120	44200	4.3%
NBA	17.8	704	830	126	44400	4.6%
DCE	10.4	726	834	108	46000	7.0%
DCM	9.14	720	828	108	46800	9.0%
Ethyl ether	4.34	658	786	128	52400	10.0%
Dioxane	2.21	672	784	112	41600	11.0%

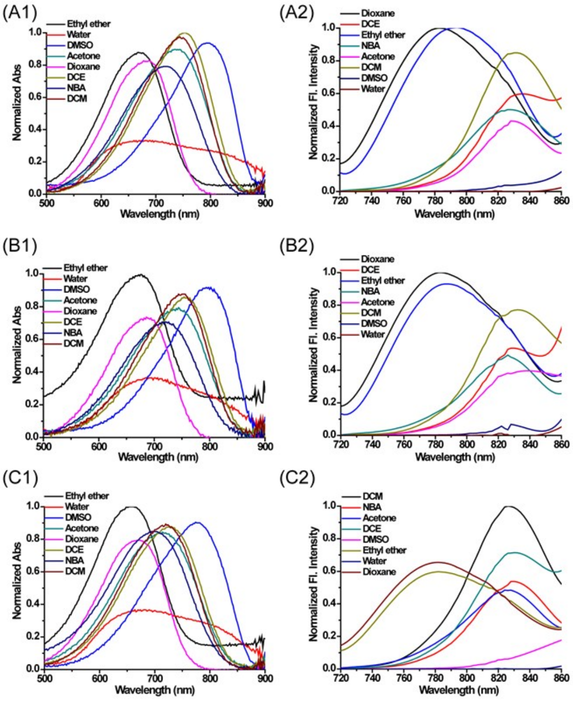

Fig. S1 The normalized absorption and fluorescence spectra of **1** (A1, A2)**, 2** (B1, B2), **4** (C1, C2) (10 μM) in eight solvents with different polarity. The excitation wavelength is 700 nm.

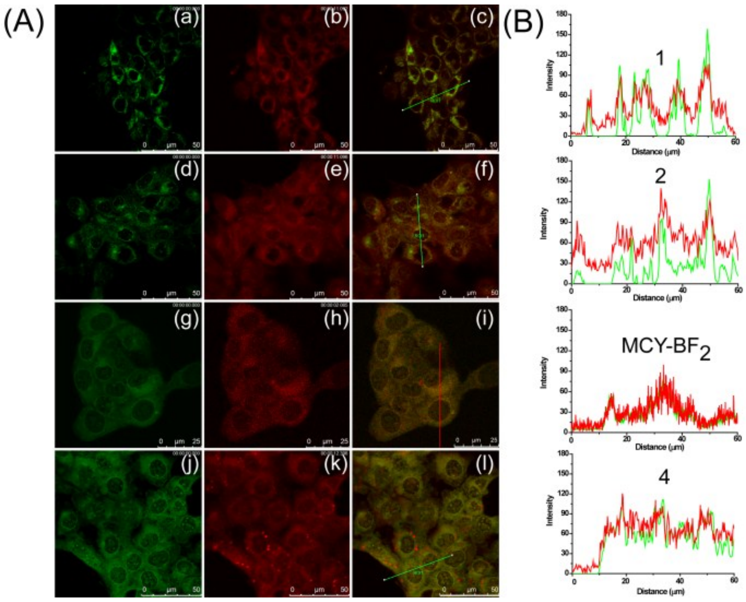

Fig. S2 (A) Confocal fluorescence images of 4T1 cells stained with **1-4** (10 μM) and **Mito-Tracker Green**. The green channels a, d, g and j represent the fluorescence of **Mito-Tracker Green** acquired by using excitation and emission windows of 488 nm and 495-550 nm. The red channels b, e, h and k represent the fluorescence of **1-4** with excitation and emission windows of 633 nm and 750-800 nm. The images c, f, i and l were the overlay images. (B) The profiles of fluorescence intensity in a line marked in c, f, i, and l.

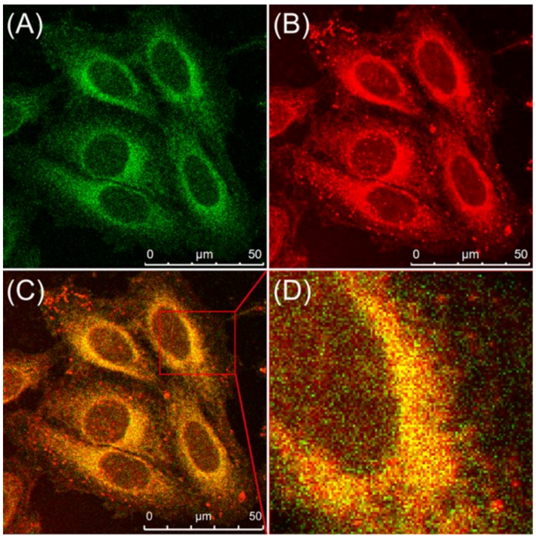

Fig. S3 Confocal fluorescence images of HepG2 cells stained with **MCY-BF$_2$** (10 μM) and **JC-1** (10 μg/ml). (A) Image of **JC-1** using excitation and emission windows of 488 nm and 510-550 nm. (B) Image of **MCY-BF$_2$** using excitation and emission windows of 633 nm and 700-800 nm. (C) is merged image of (A) and (B). (D) is the enlarged view of marked area in image (C).

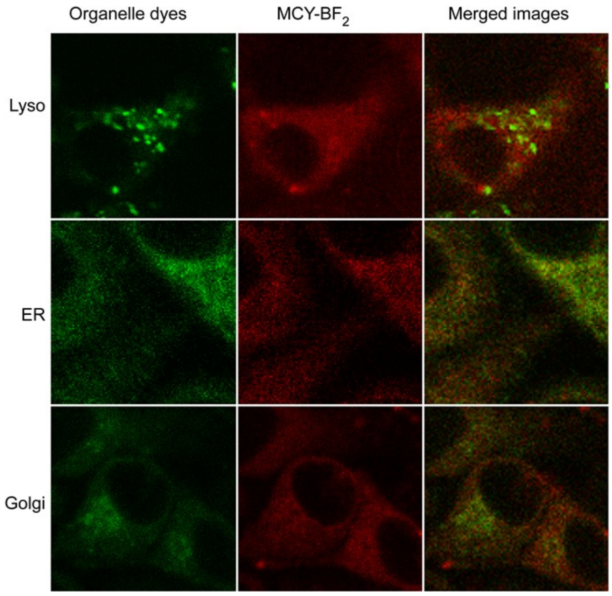

Fig. S4 Confocal fluorescence images of HepG2 cells stained with **MCY-BF$_2$** and **Lyso-Tracker Green** (0.1 μM), **ER-Tracker Red** (0.5 μM) and **Golgi-Tracker Red** (0.5 μM). Image of **Lyso-Tracker Green** was acquired by using excitation and emission windows of 488 nm and 495-550. Images of **ER-Tracker Red** and **Golgi-Tracker Red** were acquired by using excitation and emission windows of 543 nm and 580-

650 nm. Image of **MCY-BF$_2$** was acquired by using excitation and emission windows of 633 nm and 720-800 nm. The co-localization coefficient values were 0.03, 0.12 and 0.09 for lysosome, endoplasmic reticulum and Golgi apparatus, respectively.

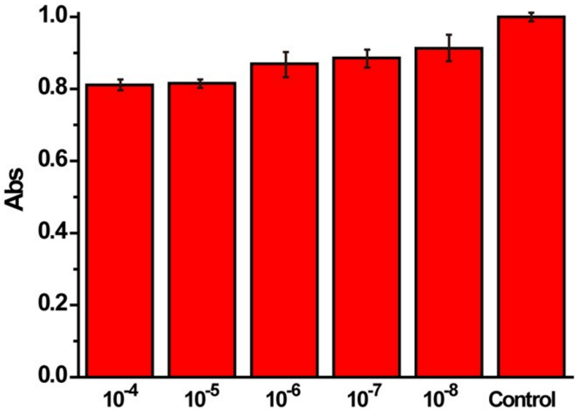

Fig. S5 The MTT experiments of **MCY-BF$_2$** under different concentrations. The IC_{50} value was 0.2 mM.

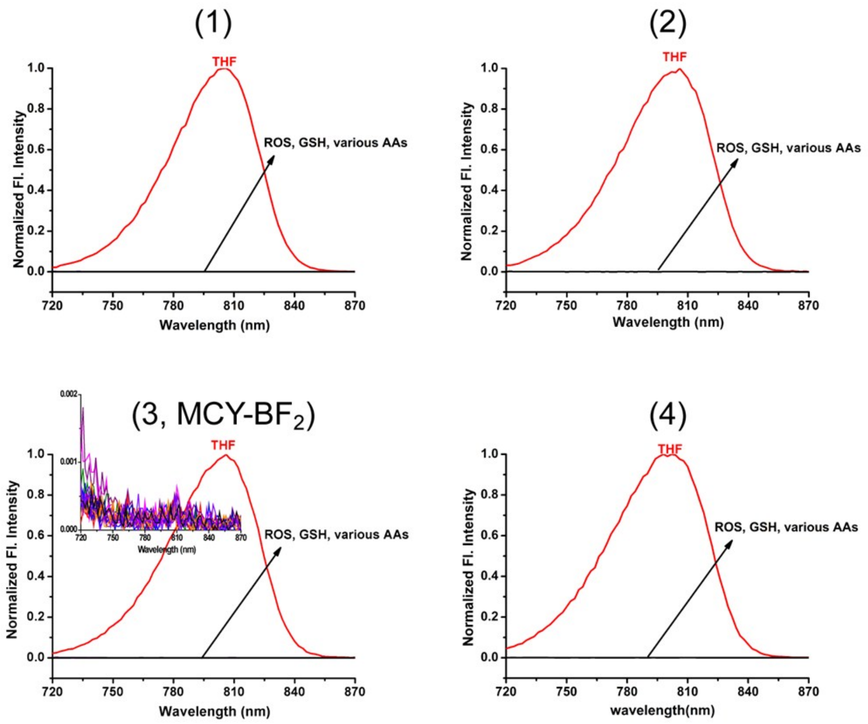

Fig. S6 The fluorescence spectra of **1-4** (10 μM) in the presence of various ROS (ONOO$^-$, $O_2^{\cdot -}$, $^1O_2$, TBHP, H_2O_2, $\cdot OH$, HClO, 100 μM), nucleophilic thiol and amino acids (GSH, Cys, HCys, Thr, Ser, Gln, Asn, 100 μM). All the species had no effect on the fluorescence of **1-4**, however, probes **1-4** showed intense fluorescence in the low polarity solvent THF.

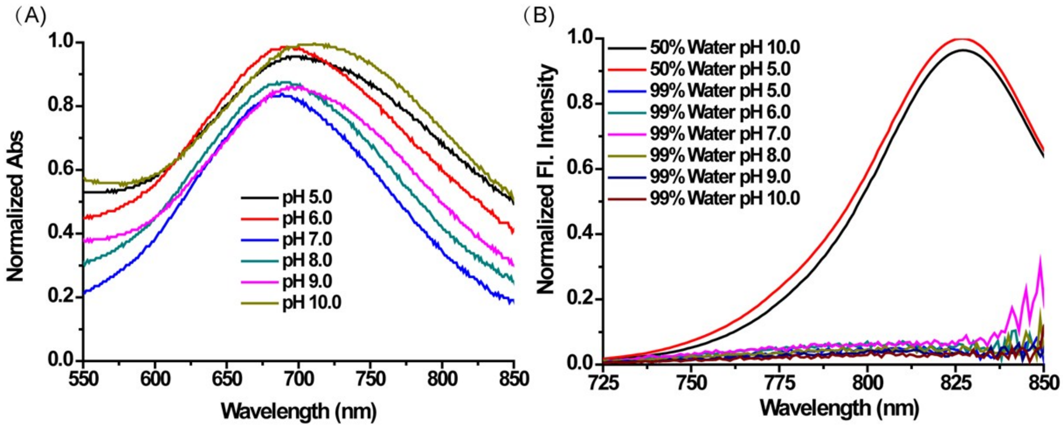

Fig. S7 (A) The absorption spectra of **MCY-BF$_2$** (10 μM) under different pH values buffer (99% water) (B) The fluorescence spectra of **MCY-BF$_2$** under different pH values buffer. The under six lines contain 99% water with corresponding pH values, and the red and black lines contain 50% water under pH 5.0 and 10.0.

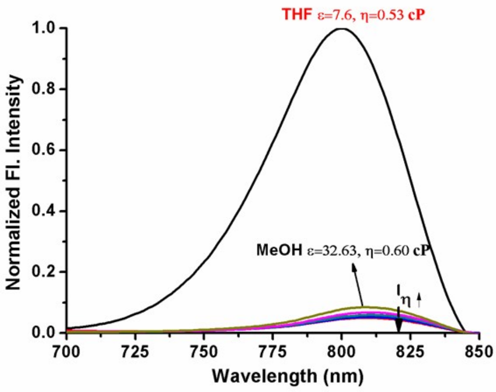

Fig. S8 The fluorescence spectra of **MCY-BF$_2$** (10 μM) in methanol-glycerol system under different viscosity. THF and methanol have almost the same viscosity (0.53 cP *vs* 0.60 cP) but different polarity (ε=7.6 *vs* 32.63).The fluorescence intensity of **MCY-BF$_2$** displayed huge difference in them. The fluorescence intensity changed little with increasing viscosity from 0.60 cP to about 100 cP.

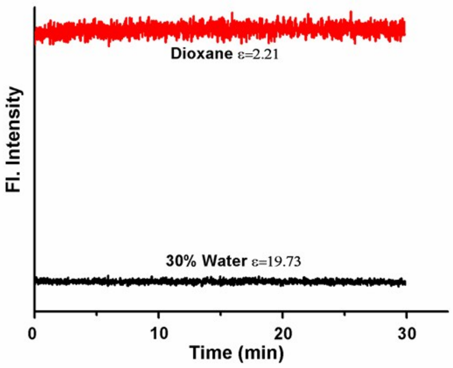

Fig. S9 The photostability experiments of **MCY-BF$_2$** (10 μM) under different polarity condition.

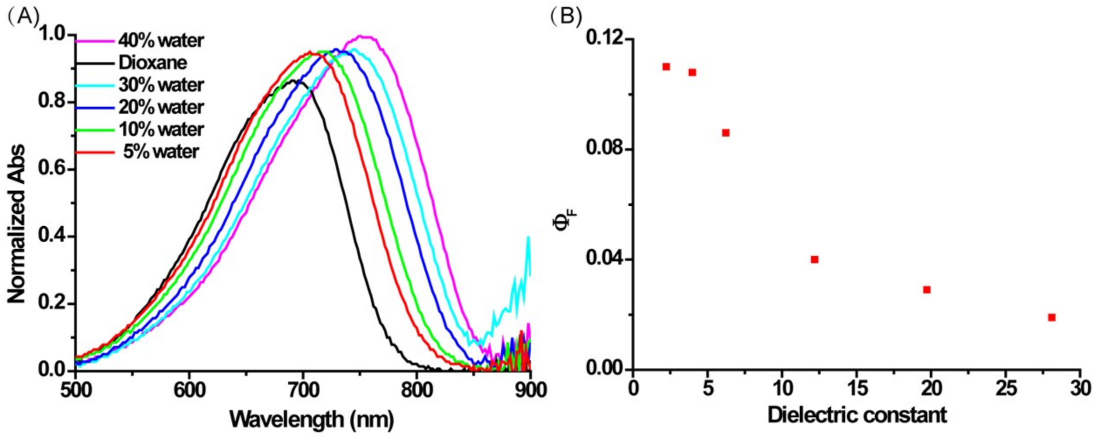

Fig. S10 (A) The absorption spectra of **MCY-BF$_2$** (10 μM) in dioxane-water mixtures (water from 0 to 40%). (B) The relative fluorescence quantum yield of **MCY-BF$_2$** in dioxane-water mixtures.

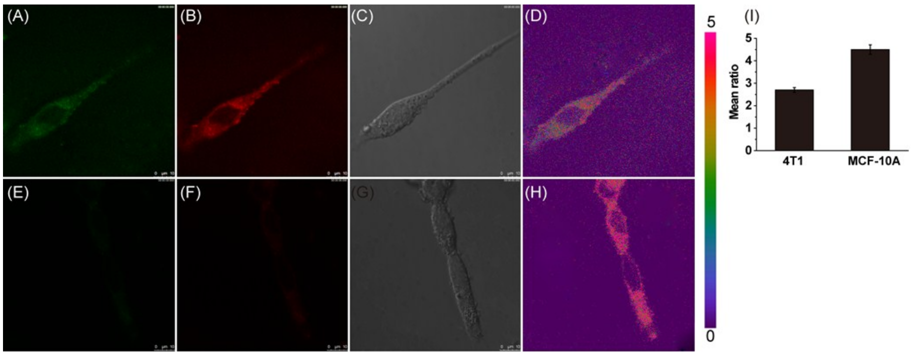

Fig. S11 The ratiometric fluorescence imaging of mitochondrial polarity in 4T1 (A-D) and MCF-10A (E-H) cell lines with **MCY-BF$_2$** (10 μM). (A) and (E) were green channels collected 760-770 nm. (B) and (F) were red channels collected 790-800 nm. (C) and (G) were bright-field images. (D) was ratiometric image between image (B) and image (A), and (H) was ratiometric image between image (F) and image (E). (I) was output of mean ratio in images (D) and (H). The mean ratio in 4T1 cells is 2.70±0.11, indicating a corresponding dielectric constant of 9.58±0.29. The mean ratio in MCF-10A cells is 4.50±0.21, which indicates the corresponding dielectric constant should be more than 30.

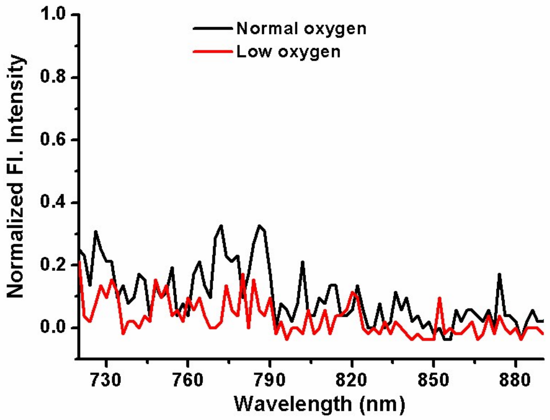

Fig. S12 The fluorescence spectra of **MCY-BF$_2$** (10 μM) in normal and low oxygen concentration solutions. Dissolved oxygen was removed with an argon gas stream for 30 min in experimental solution, then **MCY-BF$_2$** was added to the solution. The fluorescence of **MCY-BF$_2$** in this oxygen-deprived solution (red line) had no enhancement compared with that in a control solution without any processing (blank line).

Reference

1. D. Magde, G. E. Rojas and P. G. Seybold, *Photochem. Photobiol.*, 1999, **70**, 737-744.
2. D. Oushiki, H. Kojima, T. Terai, M. Arita, K. Hanaoka, Y. Urano and T. Nagano, *J. Am. Chem. Soc.*, 2010, **132**, 2795-2801.
3. H. D. Ilge, E. Birckner, D. Fassler, M. V. Kozmenko, M. G. Kuz'min and H. Hartmann, *Journal of photochemistry*, 1986, **32**, 177-189
4. X. J. Peng, F. L. Song, E. H. Lu, Y. N. Wang, W. Zhou, J. L. Fan and Y. L. Gao, *J. Am. Chem. Soc.*, 2005, **127**, 4170-4171.
5. P. Li, H. B. Xiao, Y. F. Cheng, W. Zhang, F. Huang, W. Zhang, H. Wang and B. Tang, *Chem. Commun.*, 2014, **50**, 7184−7187.
